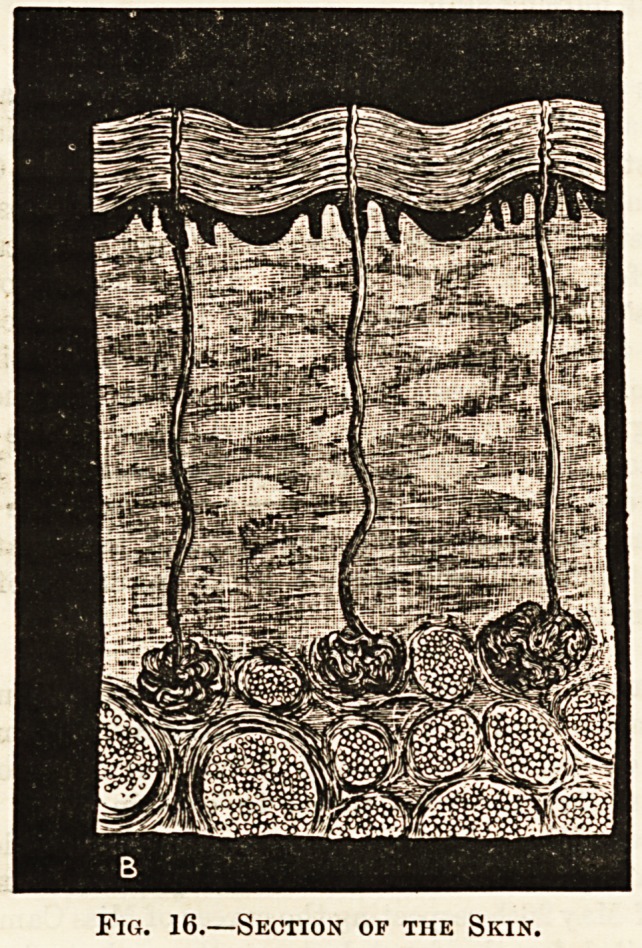# "The Hospital" Nursing Mirror

**Published:** 1900-06-23

**Authors:** 


### "The Hospital" Nursing Mirror.

Being the Nursing Section of "The Hospital."

[Contributions for this Section of "The Hospital" should be addressed to the Editor, The Hospital, 28 & 29, Southampton Street, Strand, London, W.C., and should have the word "Nursing" plainly written in left-hand top corner of the envelope.]

### Notes on News from the Nursing World.

### MORE NURSES FOR SOUTH AFRICA.

The Secretary of State for War informs us that the under-mentioned 20 nurses of the Army Nursing Service Reserve will embark for South Africa about the 26th inst.: Misses J. J. Armitstead, L. J. Attree, C. Brown, L. A. Cowley, B. Crocker, J. F. Farrer, H. Freer, G. E. Francis, M. Hay, E. Heaton-Cole, J. Mason, J. McLeod, M. J. McNair, M. C. Reilly, F. Smallwood, J. E. Smith, C. M. Theophilus, L. A. White, E. A. M. Wilson, A. Wishart. Miss Louisa Jane Attree was trained at Royal Alexandra Hospital for Children, Brighton, and at the Sussex County Hospital. She has been staff nurse in the latter institution, and since March, 1894, she has been attached to the Nurses' Co-operation. Miss M. Hay was trained at the Royal Infirmary, Aberdeen, where she was staff nurse for three years. She has also been nurse at the South-Western Fever Hospital, charge night nurse at the London Temperance Hospital, and since 1896 has been engaged in private nursing. Miss Emillie Heaton-Cole was trained at Monsall Fever Hospital and the General Infirmary, Salisbury. She has since been head nurse at the Homerton Fever Hospital, sister at the Kent and Canterbury Hospital, and night superintendent at Homerton Fever Hospital. Miss Jessie Mason was trained at the Royal Infirmary, Glasgow, where she has since been staff nurse for a year. From January, 1899, she has been engaged in private nursing.

### THE RECEPTION OF GLASGOW NURSES BY PRINCESS CHRISTIAN.

Last week, in connection with the opening of the Ruchill Hospital at Glasgow, the Princess Christian received in a semi-private way representatives of the nurses of the several nursing institutions of the city, in the Nurses' Recreation Hall of the hospital. The following were present: Mrs. Strong, matron, Royal Infirmary, and Nurses; Miss Newbury, matron, Western Infirmary, and Nurses; Mrs. Sinclair, matron, City Hospital, and Nurses; Mrs. Harben, matron, Royal Hospital for Sick Children, and Nurses; Miss Husband, matron, Maternity Hospital, and Nurses; Miss Torrance, matron, Cancer Hospital, and Nurses; Miss M'Farlane, matron, Victoria Hospital, and Nurses; Miss Adams, matron, Ruchill Hospital; Matrons and staff representing asylums; and also Miss Farrell, district superintendent, and two nurses of the Jubilee Nurses. All the matrons and nurses were in uniform. Mrs. Strong, Mrs. Harben, Miss Husband, and Miss M'Farlane wore the silver medal of the R.B.N.A.; while Miss Newbery and Miss Husband and one of the nurses displayed the armlet of the Princess of Wales' R.N.P.F. After the presentation of the matrons to the Princess the Lord Provost was requested by Her Royal Highness to say how pleased she was to meet so many nurses. Subsequently, Miss Adams, matron of the new hospital, showed the matrons and nurses over the handsome building. In the administration block there is bedroom accommodation for 200 nurses, and lavatories and bath-rooms are arranged on a most complete scale. There is a large recreation-room, already alluded to, as well as sitting-rooms for use during leisure hours, and four large sick-rooms are provided in case of non-infectious illness occurring among the nurses.

### IMPERIAL NURSING RESERVE.

The writer of an interesting article on the need of an Imperial nursing reserve in the *Morning Post* of Saturday easily shows that there are not enough army nurses. In fact, this has been proved over and over again during the present war. But the criticisms on the absence of special training in the case of members of the Army Nursing Reserve who have been drafted to the front, is answered by such a statement as that of Sir William MacCormac on Monday evening at the banquet of the Salters' Company. Of the 666 female nurses in South Africa, Sir William said, "they are incomparable"; and no complaint of inefficiency has been made by the medical authorities. Therefore, such allegations as that the nurses sent out were "professionally bewildered by the class of cases they are called upon to nurse," fall to the ground. We entirely agree with the author of the article that there "are at present far too many orderlies, and far too few nurses in the military hospitals," and any departure tending to alter this state of things would be welcomed. But it is a pity that a proposal to form an Imperial nursing reserve, to which each of the colonies should be called upon to contribute, should, in spite of a disclaimer, have been accompanied by reflections on the competency of members of the profession who have admittedly proved equal to a great emergency.

### NURSING AT BLOEMFONTEIN.

An Army Reserve Nursing Sister at Bloemfontein writes:—

"We are at present working in a school which has been turned into a hospital, and a very good one it makes. There are five sisters here altogether. We live in a cottage about five minutes' walk from the hospital. We have to do all our own housework, as we cannot get a servant for love or money, although we have been trying hard to get a Kaffir girl. How glad now we should be to see the *bête noir* of our homeland hospitals just walking in here—I mean a wardmaid. If she did not always sweep well, if at times she burnt our bacon and boiled our tea, yet now her many little acts of kindness rise up before us and keep her memory green. I miss not being able to have a bath. I never valued our roomy baths half enough. Our sisters at Cape Town are in the lap of luxury with regard to water, for they tell us that they have plenty of both cold and hot. The soldiers are brought here in a sad state sometimes off the veldt. One poor fellow had sores on him from the bites of vermin. I am on night duty—we take it in turns, a week at a time. The nights are bitterly cold, but we have no fires. We have a spirit lamp and kettle to make our tea. Lady Roberts and her daughters are living at the Presidency. She has turned a large hall into a ward for patients. I have come to the conclusion that inoculation is no protective against typhoid, for I have several cases that have been inoculated. Neither does it insure recovery, for some who were inoculated have died. Everyone is very kind to us, and the doctors and officers do all in their power to make us happy and keep us well."

### A NURSE IN THE SLOUGH RAILWAY CATASTROPHE.

One of the victims of the disastrous railway accident at Slough on Saturday was, we regret to learn, a nurse, Miss Rose Stanley, who has just returned from nursing wounded soldiers in South Africa. Miss Stanley was in a front corridor carriage of the express, and was thrown with great violence from side to side, her head and back receiving heavy blows. When she was extricated she was unconscious, and it was found that her back was injured. She was placed under medical care, and, at her own entreaty, was allowed to proceed to Bristol to her home, to which she was conveyed in an ambulance. A sad home-coming indeed!

### NURSING THE VICTIMS OF THE SLOUGH ACCIDENT.

"On Saturday afternoon, about ten minutes past three," writes one of the nurses of St. Mary's Hospital, "our casualty porter came in and told us there had been a smash up on the line at Slough, and that some of the wounded were on their way to us. Presently three men were brought in on stretchers, and almost before we had time to get them settled comfortably more came, and more, until by about a quarter to four the place looked like a miniature battlefield, and we felt we were really receiving our baptism of fire. One of the sufferers said to me, 'Nurse, I had the chance of going to South Africa, but somehow did not relish the idea of being bowled over, and now here, near London, I have got my knock; a good thing for us we cannot see ahead. God be thanked for that!' The patients were all men, and they were taken into the wards as quickly as possible. Offers of assistance were simply showered upon us from all sides. We have never had such quiet sufferers, not one complaining, and all far more concerned about their friends than about themselves. One poor fellow had been going to meet his *fiancée*. His nerves seemed greatly shaken. He did not say a word, but the big tears kept coming. However, as soon as we solved the problem that troubled him, and a telegram was sent, he felt better. One was brought in unconscious, and died almost immediately. Sixteen others were admitted, and are all doing well. A poor old gentleman had no wound of any kind, but seemed just dazed. He could not keep still, but walked about, up and down, in and out, and we made him come into our room, sit in an easy chair, and sip a little brandy and soda. After a time he was able to go to his home."

### THE NEW NURSES' HOME AT PLYMOUTH.

The ceremony called the laying of the foundation stone of the new Nurses' Home at the Royal Albert Hospital, Plymouth, which was performed by the Princess Henry of Battenberg a few days ago, was really the inauguration of the extension of building. The home is unpretending in appearance, and is not, like the hospital, constructed of Plymouth limestone, but of brickwork with Hamhill stone and white brick dressings. The Tudor style of architecture is observed. The heating is obtained by hot-water pipes so as to obviate the use of fires. A covered bridge runs from the main floor to that of the hospital, and there is thus an uninterrupted communication between the two. Accommodation is provided for 20 nurses, and there are 14 bedrooms, six cubicles, sitting-room, bath-room, and lavatories. A dining-room has been constructed by partitioning off the west end of No. 1 ward, now used as the chapel.

### MRS. GLADSTONE AND CONVALESCENT HOMES.

Mrs. Gladstone, the great lady whose remains were appropriately laid to rest on Tuesday by the side of those of her great husband in Westminster Abbey, took a keen interest in nursing movements. It was mainly through her instrumentality that the free convalescent home movement was initiated. In her welcome visits to the London Hospital during the epidemic of cholera in 1866, Mrs. Gladstone strongly felt the need of provision for the convalescents, and for the orphans of those who suffered from the disease. Starting a small home for the latter at Clapton, she ultimately founded the orphanage at Harwarden; while as to the former, she opened a convalescent home at Snaresbrook. This was soon transferred to Woodford, and was carried on there until last year. Mrs. Gladstone's intention was that the home should specially serve for the use of the convalescents from the London Hospital, and from the outset the system of privilege tickets was discarded. In 1876 her famous husband pleaded the cause of Mrs. Gladstone's convalescent home at the Mansion House.

### AN INSPECTOR ON NURSING IN WORKHOUSES.

Mr. P. H. Bagenal, an inspector of the Local Government, made some remarks of importance the other day in reference to a request from the chairman of the Loddon and Clavering Board of Guardians. There have been difficulties in Heckingham Workhouse, owing to the clashing of authority between the master and matron and the nurse, resulting in the resignation of the latter and of a new appointment. Mr. Bagenal having expressed a hope that friction will be avoided in the future, went on to point out the advantages of the order of the Local Government Board forbidding the nursing of the sick by pauper inmates of the workhouse. He said that in considering the position of nurses in workhouses, he wished to make it clear—and he was very strong on this point—that in regard to the care of the sick the nurse was responsible only to the medical officer, but that in all other matters connected with the discipline of the workhouse she must be under the control of the master. "They must have the master at the helm, or else there would be no supervision whatever. At the same time the master and matron must remember that in their nurses they had a new and professional class of persons to deal with who were entitled to every possible consideration, and unless there was desire and an effort on both sides to make things go smoothly he was afraid it would be found very difficult to go on. The whole question hinged upon the desire of the officials to work harmoniously." Mr. Bagenal put the case very fairly from the official point of view. So long as the existing system obtains, it is clear that peace is only possible by the exercise of forbearance on both sides.

### LIBEL ON A NURSE.

Damages to the extent of £40 have been awarded to Mrs. Sarah Wright, a nurse with 17 years' experience, who brought an action for libel against Mr. Richardson, of Selly Park, Birmingham. The libel was contained in a letter written by Mr. Richardson in reply to one addressed to his wife asking for a reference in regard to the capacity of Mrs. Wright. He gave a "reference," in which he charged the nurse with bad temper, vulgarity, impudence, inattention, and dubious qualifications. It was pleaded that this letter was privileged, but as the jury decided otherwise, and the evidence proved to their satisfaction that the nurse was not the kind of person represented by Mr. Richardson, the damages given are lenient. Two doctors, under whom Mrs. Wright had nursed, spoke in favour of her, and Mr. Richardson's own evidence in support of his allegations was not impressive. He said that "she neglected the patient, inasmuch as she did not supply the necessaries, such as beef-tea and gruel. She wasted her time 'by staying in the kitchen, playing the piano, and reading the paper.' She was impudent, in that she went to a clergyman and arranged for the child to be christened and the church to be heated. She borrowed coal when he told her not to, and finally on one occasion, when a gentleman was visiting, she 'joined in the conversation, and monopolised it to such an extent that neither me nor my wife could get a word in edgeways.'" These are very shadowy foundations for an attack which, if it could have been justified, would have rendered it practically impossible for Mrs. Wright to pursue her profession.

### EXAMINATION AT LAMBETH INFIRMARY.

The result of the nurses' examination at Lambeth Infirmary was exceedingly satisfactory. Out of the eighteen candidates presenting themselves only three failed, and the failures were all in theoretical and not in practical work. The examiner, Dr. Hector Mackenzie, wrote to the matron after the examination congratulating her on the uniform excellence of the practical demonstrations of her nurses.

### WARWICK NURSING ASSOCIATION.

The work of the Warwick District Nursing Association during the past year has been of a very satisfactory character. The number of cases was 155, the number of visits paid was 6,123, and financially the balance is on the right side. The Countess of Warwick, who presided at the annual meeting, said that the nursing had become more widely known and better appreciated than ever before, and that the members of the association had reason to congratulate themselves upon the progress it was making. But she affirmed that there was room for a great deal more energy, and she exhorted the ladies of Warwick to join more generally in the task of collecting funds. At present there are only two nurses. Lady Warwick states that a third is wanted, and will be engaged as soon as the money can be found. Dr. Gardner, who expressed his belief that the efforts of the nurses had much decreased the number of persons in receipt of parish relief, declared that the Board of Guardians ought to assist the association.

### THE HENRIETTE RAPHAEL MEMORIAL.

The large nurses' home which will provide 200 of the Guy's Hospital nurses with every comfort and convenience that modern thought can devise, is making rapid progress. It occupies a site to the left of the entrance into the recreation ground, or the "Park" as it is called. When completed the home will be five storeys high. It is now a hive of busy workmen; the corridors are represented by bridges of loose planks, between brick walls about three parts built. Running at right angles with the home is the block, also in process of building, for the new laundry. The "Park" is a pleasant spot. Part of it is devoted to patients, and part is reserved for nurses, who take every advantage of the opportunities it affords for rest and quiet.

### THE NURSES OF BRISTOL ROYAL INFIRMARY AND THE PENSION FUND.

The committee of the Bristol Royal Infirmary, recognising the exceptional advantages offered to nurses by the Royal National Pension Fund for Nurses, have decided to pay part of the premiums for all their sisters and private nurses. This, it is hoped, will be an encouragement to the nursing staff to join the Pension Fund. The new arrangement will come into force in October. Nurses at the Bristol Infirmary, in addition to gaining a good general experience in nursing, have the opportunity of obtaining training in midwifery and massage free of expense. Now that the new nurses' home is completed the committee intend to increase the number of their nursing staff.

### THE NEW CENTURY BAZAAR.

The New Century Bazaar, held last week at the Portman Rooms, Baker Street, was of considerable interest to a large number of nurses. S. Ellen Orme, late matron of the London Temperance Hospital, Hampstead Road, was hon. secretary, and the president (the Hon. Mrs. Eliot Yorke), and members of the Nurses' Total Abstinence League helped, and contributed to the stall of the Woman's Total Abstinence League. The Nurses' Total Abstinence League has made great progress lately, and evidences of the good influence exerted by members is not lacking. Many nurses engaged at the infirmaries and workhouses have had the satisfaction of seeing some of their patients abjure alchohol, and become sober members of the community.

### SHORT ITEMS.

Lord Loch, president of the Colonial Nursing Association, died on Wednesday.—Miss Annie Cameron, Sister of the Army Nursing Service, who is now on duty in Egypt, writes to us to explain that there are two Miss Annie Camerons in the service of the Army, and that the details published in our issue of May 26th respecting the career of Miss Cameron, who sailed for the front last month, apply to herself. —The Helen Prideaux Memorial Scholarship, value £50, will be awarded in July, 1900, to a graduate of the London (Royal Free Hospital) School of Medicine for Women. Candidates must be registered medical women of not more than three years standing on June 1st, 1900, and must send in to the trustees, under cover to the secretary, an essay on some medical subject, on or before July 15th, 1900. The subject chosen by each candidate may be connected with any department of medical or surgical practice. In estimating the relative value of the essays, the trustees will attach special value to the evidence afforded of clinical work, and of direct and personal experience.—The annual meeting of the Working Ladies' Guild was held last week, by the kind permission of Lady Susan Leslie Melville, at 48, Belgrave Square. The guilds' work among distressed ladies has largely increased during the past year, £270 more having been given away to cases of distress, sickness, and in grants for education. Subscriptions and donations were larger, but not in proportion to the number of cases of urgent distress needing immediate and permanent relief. £4,157 has been distributed in the relief of distress, and in payments for work.

### Lectures on Nursing for Probationers.

By E. MacDowel Cosgrave, M.D., &c., Lecturer to the Dublin Metropolitan Technical School for Nurses.

### IX.—THE SKIN.

The skin is made up of two layers—on the outside is the epidermis, inside is the derma. The epidermis is the thin structure which is raised by a blister. The thickness of the entire skin varies in different parts, but may be as much as a quarter of an inch; this can be seen in the incision made in an operation. The surface of the derma is not smooth, but is raised up into little finger-like processes, called papillæ; the epidermis is thinner over these papillæ than in the hollows between, so they are not as prominent in appearance as in reality; they can, however, be seen on the finger-ends, where they are in great number, the fine lines that give the characteristic markings to the tips being made up of rows of papillæ. On other parts of the body they are larger but much fewer; when the skin is chilled they become prominent, and give rise to the roughness known as "goose-skin."

Fig. 16.—Section of the Skin.

The derma is chiefly composed of white connective tissue, in the deeper parts are muscular fibres and elastic fibres, and there is a good deal of fat; blood-vessels and nerves also occur—they give off branches to the papillæ. The lowest white fibres of the derma are connected with the sheaths of the muscles beneath; as the threads run in every direction they form only a loose attachment (as is shown when the skin is pinched up), and so the movements of the muscles are not interfered with.

In each papilla is a network of capillary blood-vessels, and in most is a nerve ending in a peculiar round or oval knob. These nerve endings are the seat of feeling, and convey to the brain such sensations as touch, temperature, and pain.

The lower part of the epidermis consists of a layer of cells, which are supplied with nourishment by the capillary blood-vessels in the papillæ. These cells have the power of dividing, and so giving off new cells. The rest of the epidermis is made up of the cells so given off; they are round and soft when first made, but gradually get harder and flatter until towards the surface they are merely dry scales. It is of these scales that the horny layer of the epidermis is composed. Any irritation causes the deep cells of the epidermis to give off new cells more rapidly and in greater profusion; so, when the hand, for instance, is used for hard work, instead of the epidermis getting worn off and exposing the nerves, it actually gets thicker and protects them.

The skin contains sweat glands, fat—or sebaceous—glands, and follicles, from which hairs grow.

The five chief uses of the skin are: (1) To support the tissues of the body. (2) To protect. The epidermis protects the nerves in the papillæ; if the bare nerves are touched, as, for instance, on a blistered surface, nothing can be felt but pain; the epidermis forms a cushion, preventing direct contact and yet allowing shape, size, hardness, heat, &c., to be recognised. (3) To act as an organ of touch. This function depends on the nerve endings in the papillæ; the more closely they are placed the more accurate the feeling. In the finger tips they are closely crowded together. (4) To regulate the temperature. This is done by the movements of the blood and the action of the sweat-glands. When the temperature is raised the hot blood rushes to the skin, and sweat pours from the glands; the evaporation of this moisture cools the blood in the skin, and this cooled blood returning into the body cools the deeper structures. (5) To act as an organ of excretion. Both the sweat glands and the sebaceous glands give off waste materials.

Hairs grow from a bulb at the bottom of a little tube or follicle; they pass through the skin in a slanting direction; one or two fat glands open into each follicle, and muscular fibres are attached close to the bulb; it is the contraction of these muscles through cold or fright that causes roughness of the hair.

Inflammation of the skin is marked by four prominent symptoms—redness, heat, swelling, pain. The structure of the skin explains how each of these is caused. Redness depends upon fulness of the blood-vessels from relaxation of their walls. Heat is caused partly by the extra amount of blood and partly by too great destruction or burning of tissues. Swelling is caused partly by the over-filled blood-vessels and partly by the uncoloured part of the blood escaping through the distended vessels into the surrounding tissues. Pain is caused by stretching of and pressure on the nerves. If a small portion of tissue dies, a boil results, the dead tissue is cut off from the living tissue by formation of pus or matter; the dead tissue and pus gradually work up in the skin until they escape through it.

When a number of small pieces of tissue die, the intervening tissues may also be killed and an anthrax result. This may spread until it is a very large size. In an abscess there is a limiting membrane formed round the matter; this prevents spreading. In cellulitis, erysipelas, &c., there is no such limiting membrane, and the inflammation and formation of matter may spread rapidly through the connective tissue and cause extensive destruction.

Wounds of the skin may be incised, contused, lacerated, or punctured. An incised wound or a clean cut may heal by direct union, or, as it is sometimes termed, by first intention. If the two sides are placed together and kept quietly in position, the surfaces get glued together by white blood corpuscles, and the cut tissues reunite. In contused (bruised) and lacerated (torn) wounds the tissues are too much injured for this, so they have to heal by granulation, new tissue growing up from below and filling the wound; this new tissue then contracts, drawing the sides together, and itself forming the white scar tissue. Punctured wounds are very dangerous, as they are hard to clean, and if dirt gets into them blood poisoning is liable to follow.

### Nursing in America.

By an English Nurse.

### II.

I had taken up my abode on a Wednesday, and for four days I lived the life of a free, independent, and unfettered American citizen, being even—for the first time in my life—the proud possessor of a latch-key.

### The Cases.

On the Sunday morning my first summons came, a little, truth to tell, to my disappointment, for I had made an engagement for the river for the afternoon, and had no means of letting the friends with whom it was made know that I could not come. However, it was my first case, and I certainly was not going to refuse it. It turned out to be a doctor's wife, and nothing could exceed the kindness and courtesy I received from both doctor and patient. Indeed, their kindnesses extended to long after I left their house, and more than once—in fact, pretty frequently during the rest of my stay in Boston—I was rung up to ask if I could not come out to lunch, or dinner, or to go driving or sleigh-riding. After that case three more followed in rapid succession. My cases were entirely among the professional classes, though in America you must be prepared to nurse anyone and everyone. Everyone seems to have money, and when the necessity arises, the man who drives a waggon, and the joiner's wife seem as able to pay for the services of a nurse as people in quite different positions. As far as the mere nursing went, it was pretty much what it is at home. Perhaps the restless, nervous American temperament wants a little more waiting on—a little more "fussing over." Medicines are given more frequently than we as a rule are used to—every two hours perhaps for one medicine, and three or four times a day for another, being the usual thing.

### The American Quack.

It was in nursing my third case that I made my first acquaintance with an American quack. Previously the doctors, whether surgeons or physicians, had been as clever and as polished as those on our own side of the Atlantic. My patient was an old man of eighty-five with pneumonia. A specialist had been called in, but neither he nor the doctor attending the case, a particularly skilful man, could give any hope. It was then that one of the daughters suggested a "Dr." Jones, whom she alleged had performed some wonderful cures by hypnotism, and after discussion among the family it was decided to call him in. The doctor attending the case looked blank when he heard, but, as he said afterwards, What could he do?

"I'll go now," he said in an undertone outside my patient's door, "but will come back as soon as he goes. You must remain in the room, and see that he does not practise any experiments on the poor old man. If he does, stop it and send for me; I can be here in two minutes."

After a little while Dr. Jones arrived, a tall dark handsome man, with a suave manner and a strong face, whom I could quite imagine impressing weak hysterical women.

"Ah!" he remarked, proceeding to examine the patient's chest. "Yes, pretty bad! Pulse 130. Respiration 40. Temperature—what did you say the temperature was?" turning to me.

"102·8 deg.," I answered briefly.

"Yes, still I think I am in time! Yes, I think I am! I will first proceed to get the pulse better—so." For some five minutes he stood, both hands placed over the patient's heart, gazing steadily at him.

"Capital! capital!" he kept muttering to himself. "The beat is 120 now—still it improves—now it is 110." Then after a little while: "Now it is 100. Your father will, I trust, live," he said to the girl standing crying quietly at the foot of her father's bed.

It was horrible, for it seemed to me the patient grew worse all the time, and it was with the greatest difficulty that I asked civilly if he would kindly allow me to give the brandy now ordered by Dr. ——.

"Oh, yes, certainly, my dear young lady," he said, in a patronising tone, "of course it will not assist in the least degree, but you are quite right to carry out the order given you. Afterwards, will you get me a basin of water, as I intend now to reduce the temperature."

He took the basin and placed it on a chair beside him, whilst I resumed my place at the foot of the bed.

"I am about," he went on, "to transfer that water by induction into the patient's veins, from which it will be carried through the heart into the arterial circulation and so reduce the fever." For another half-hour the farce went on, and then he rose to go, and all the way downstairs I could hear him assuring the family he had reduced the pulse to 100 and the temperature to just over 90 deg., and now he hoped the patient would live, though had they called sooner he would have felt surer.

Just as soon as he went I took my patient's pulse—it was 135; I did not trouble to take the temperature. Next day my patient died.

My fourth case brought me to within a few days of Christmas, and as I had been doing pretty well and everyone assured me Christmas was the time to see New York, I proceeded to pay a fortnight's visit to that city.

### Specialisation in Nursing.

Whilst in Boston I had paid several visits to, and heard several lectures at, the City Hospital by the kind invitation of the superintendent, to whom one of my friends had given me a letter of introduction. I had also gone over the Massachusetts. Everything, especially as regarded all pertaining to surgery, is very perfect in both hospitals, but what struck me most was how on earth the nurses managed to get an allround training. Everything is so specialised. One hospital has no children's ward, the other a very small one, and when I asked a nurse who had been there two years how they treated their hip joint disease cases (I had been a long time in the children's ward, and was specially interested in children) she said she had never seen a case. Neither hospital has either an obstetric or an eye ward, and diseases of the throat and ear go elsewhere. The City hospital has an infectious disease block, or rather blocks, for they cover a large area, but the other hospital has none. Yet these hospitals, like all others, turn out their nurses full "graduates," able to nurse any and every case, in many instances far from the surveillance and help of doctors.

### An Ideal Hospital.

In New York, somewhat to my friends' disgust, I insisted on "doing" one or two hospitals, in addition to the regulation stores, churches, and theatres.

Bellevue was fine, but in St. Luke's, for the first time, one found one's ideal hospital. The present building is only three or four years old, and everything—building, sanitation, methods of heating and change of air, means of isolating completely every single ward from all the others, operating rooms, waiting rooms, and recovery rooms—are simply perfect. In the operating theatre every article in use is glass, and everywhere there is neither hole nor corner where dust can hide, floors rounding into walls, and walls in turn curving nto ceilings. Unfortunately, I was shown over by the rawest of new probationers, so I could find out nothing about the working. The superintendent and the assistant-superintendent are both clergymen, which to one accustomed to English ways, seemed singular; but the hospital was founded in old days down by Fifty-third Street, and carried to its present pitch of excellence by the exertions of an Episcopalian clergyman who devoted his whole life to the work. The very building shows the same influence, as, immediately on entering, right before you, approached by six steps, lies the chapel, the light from its stained windows filtering through a woodwork screen on to the white marble pavement of the entrance hall.

"If you had told me you wished to go over it I would have given you a note to the matron, which would have secured you a more interesting guide," my friend and hostess's doctor remarked a few days later.

"Yes, it is as you say a beautiful building and a superb site. Did your guide take you up to that window from which you can view the whole of the city on one side and miles up the wooded banks of the Hudson on the other?"

"Oh, yes, she was very good; she took me to most places, even to the kitchens—one where all the cooking is done, and the other where nurses are taught invalid cookery."

By the way, in some respects the American nurse is in advance of her English sister; in addition to cookery she is taught during her training to dispense, to shave, to test, and to administer ether.

Dr. G. gave me his opinion on the chances of obtaining work in New York pretty freely: "Nurses—they are simply a pest here—we are overrun with them. They are turned out every year by the hundred, and they come worrying every man with a brass plate up with applications for employment."

### Presentation.

Miss Alice South, after fifteen years' connection as matron of the rebuilt and reorganised hospital for Hastings, St. Leonards, and East Sussex, has resigned her charge and opened the Imperial Nursing Association at 57, Marina, to provide the best trained nurses and to receive patients. To mark their appreciation of the valuable and conscientious services of Miss South, and in grateful recognition of her kindly care of all under her charge, the house and general committees of the hospital recently presented her with an illuminated address, a service of table silver, and a handsome ormolu clock and pair of candelabra.

### TRAVEL NOTES AND QUERIES.

Normandy and Paris for a Week (Tiny).—Why not start from Caen instead of Havre, so as not to go twice over the ground? Newhaven to Caen direct, first-class, single, £1 5s.; second, £1 1s. Hotel St. Barbe at Caen. Cycle next day to Trouville, 25 miles. Stay at the Plat d'Or or the Louvre. Every place dear in the season. Ten miles on to Honfleur, a much cheaper place. You would do better to stay at Honfleur, and coach or cycle to Trouville. Stay at Hotel de la Paix. From Honfleur cross to Havre by the little steamer, and there take boat to go up the Seine to Rouen—all the way, if you like; but it would be nice to stop at Caudebec two or three days. Hotel de la Marine very reasonable. At Rouen stay at the Hotel de la Poste in the Rue Jeanne d'Arc. For Paris everything will be horribly dear. Hotel de Bourgogne, 8, Rue Duphot, is generally reasonable, but terms will be largely increased. Mesdames Mansfield and Townsend, 157, Faubourg St. Honoré, would take you for a week, I think. The Hotel Britannique is also reasonable, but be prepared for terms to be doubled.

Normandy and Brittany (Magda).—You are trying to arrange too much. You will do better to visit either one or the other (not both) in the time you have at your disposal. If Normandy, I advise the following route: Steamer to Caen, from which visit Bayeux and Falaise; then to Lisieux. When leaving these, start by the first train for Evreux, which will give you time to see the cathedral and bishop's palace. Arriving in Rouen late, stay a few days there and a few days at Caudebec, which will enable you to see the Seine. Take the boat home from Havre. If you choose Brittany, take return tickets to St. Malo, sleep two nights at Dinan. On to St. Briem, from which diligence to Pattapot. Again diligence to Lamion, staying at each place as well as Perros-Guireo as you like. Train to Morlaix, with a week's stay. From there visit St. Pol de Léon and Roscoff. Return (breaking the journey at Guingamp or Lamballe) to Dol, thence to the Mont St. Michael, and home viâ St. Malo.

### Death in Our Ranks.

A correspondent, writing on the death of Sister Kate Clayton at Bloemfontein from enteric, says: "She was probably better known in the nursing world as Sister Collins, having been for many years sister at St. Helena Home, Grove End Road, London. She was an early member of the R.B.N.A., and had served on the General Council. She was trained at the General Hospital, Cheltenham, and at the Shrewsbury Infirmary, and then entered the St. Helena Home. She had a wide and varied nursing experience, which she turned to the fullest account. She was exceptionally clever, both as a surgical and as a medical nurse, and very devoted to her work, inspiring her patients with perfect confidence in her professional skill, while winning their affection and respect by her sympathy and untiring care. Sister Collins held a diploma as midwife, and it was in that capacity that she went to St. Michael's Home at Kimberley, to work under Sister Henrietta. There she endured all the trials of the siege, and took part in nursing the soldiers. She was asked to transform the Christian Brothers' School into a military hospital, which she did to the satisfaction of the authorities, and subsequently, when Lord Roberts sent for more nurses to Kimberley, she was among those selected to go. There she worked devotedly till she was called away."

### Appointments.

Station Hospital, Gibraltar.—Miss Evelyn Alder has been appointed as an Army Nursing Sister. She was trained at the Royal Infirmary, Bristol, and holds certificates for massage and electricity and the L.O.S. During the last two years she has been engaged in private nursing at the Victoria Institute, Bournemouth.

Stroud Hospital.—Miss Margaret Russell has been appointed Lady Superintendent. She was trained at University College Hospital, London, and the Royal Infirmary, Edinburgh. She has since been assistant superintendent at the Kent Nursing Institute, superintendent of the Stroud District Nursing Association, and lady superintendent of the Swansea and South Wales Nursing Institute and Private Hospital.

### Minor Appointments.

Royal Hospital for Sick Children and Women, Bristol.—Miss Florence Weddall has been appointed Charge Nurse. She was trained at St. George's Infirmary, Fulham Road, and has since done temporary duty at the Cork Street Hospital, Dublin, and at the Nurses' Cooperation, London.

Birkenhead Union Infirmary.—Miss Margaret Beard has been appointed Superintendent Nurse. She was trained at Chorlton Union Infirmary, and has since, for six years, been superintendent of the lunatic, epileptic, and infirm wards at Chorlton Union Workhouse. She holds the L.O.S. certificate.

Exeter Sanatorium.—Miss L. Tinsley has been appointed Charge Nurse. She was trained at the Borough Hospital, Wolverhampton, and has since been nurse at the Homerton Eastern Hospital for three years, and charge nurse at the Borough Hospital, Kidderminster, the Sanatorium, Middlesbrough, and the Marland Hospital, Rochdale.

Victoria Hospital, Woking.—Miss Wilhelmina Redmayne has been appointed Staff Nurse. She was trained at Mile End Infirmary, and has since been nurse at the Branch Seamen's Hospital, Royal Albert and Victoria Dock, and charge nurse at South Shields Hospital.

City Fever Hospital, Birmingham.—Miss M. L. Minto has been appointed Ward Sister. She was trained at Paddington, and has since been staff nurse at Camberwell Infirmary.

Tunbridge Wells General Hospital.—Miss M. E. Lander has been appointed Sister. She was trained at the General Hospital, Wolverhampton, and has since been staff nurse at Hove Dispensary and Hospital.

Taunton Workhouse Infirmary.—Miss Gertrude A. Please has been appointed Superintendent Nurse. She was trained at Chorlton Union Hospital, and has since been charge nurse at Newcastle-on-Tyne and Warrington.

### The Nursing of Orthopædic Cases.

Clinical Lecture delivered to Nurses at the City Orthopædic Hospital by NOBLE SMITH, F.R.C.S.Ed., Surgeon to the Hospital.

THIS lecture consisted of a demonstration of the application of splints in the treatment of club-foot and bowed legs.

THE APPLICATION OF SPLINTS FOR CLUB-FOOT.

In the early stages of the various forms of club-foot much good may be done by manipulations alone, but the effect is far more satisfactory if the improved position is maintained by means of splints. The application of these splints is not quite so easy as the nurse may imagine, and as a rule at this hospital the surgeon carries out this part of the work. The nurse, however, should understand the procedure, and be ready to apply or reapply the splint if required.

The splints we use here for this purpose are made of hoop-iron, covered with some soft material and padded. They can be bent into any desired position.

The foot should be firmly moved by the operator's hands into an improved position, and the splint attached upon the side from which the foot bends. Three strips of adhesive plaster, one inch in width each, are used, one attaching the splint to the anterior part of the foot, one from the heel, and the third round the child's calf; then a bandage is to be applied outside. The plaster ensures the retention of the splint in the correct position, and prevents it from sliding round the foot.

After a few days, or perhaps a week, these splints can be reapplied with the foot in an improved position, and so by degrees we are able to overcome the deformity. Directly the tendon of a contracted muscle appears tense, it is our custom here to divide it, and then to place the foot in as good a position as possible. After the cut tendon has healed, the stretching process by means of the splint is continued until perhaps another tendon appears to be prominent and offering impediment to further improvement in position, when this tendon is also cut. By this persistent and continuous treatment most perfect results are obtained, and we seldom if ever have to resort to osteotomy, or other severe operations. In considering this subject it should be realised that when a club-foot is cured by this gradual method the bones are brought to assume their correct shape and the cure is perfect, whereas after osteotomy the result is at best a mutilation. In place of the metal splints we sometimes use plaster of Paris bandages, and nothing can be better if the surgeon and the parents do not mind the extra trouble of removing and re-applying them sufficiently often. In all cases we must be careful to avoid producing sores. Not only are the sores painful to the patient, but they seriously retard the progress of treatment, because it is obvious that the sore will be produced exactly where we want to exert pressure in the future. In applying a splint we should always have the foot held in contact with the splint in its whole length before putting on the adhesive plaster; that is to say, we should not attach the splint to the calf of the leg, and then force up the foot to the lower part of the splint. The latter procedure is apt to wrinkle the skin and produce sores. If a few hours after applying a splint the child shows evidence of pain, the nurse should at once remove the splint and reapply it with less pressure.

SPLINTS FOR BOWED LEGS.

It is frequently stated that a child will grow out of bowed legs, but if you will observe the legs of adults walking about the streets you will see how many of them have grown up with this deformity persisting. I have heard it stated over and over again that splints have failed to straighten bowed legs, and that it has been necessary to cut the bones. The reason of this failure is a want of skill in applying the splints. I am referring to treatment in children under ten years of age. Another excuse for not using splints is that, although the surgeon applies them at the hospital correctly, the parents fail to reapply them in the right position afterwards. This may be quite true with regard to splints as usually applied. They are commonly attached by a roller bandage from the foot to the knee. By this method it is difficult enough for the surgeon to bandage the leg effectually, and certainly the parents of the patients will not be able to do so.

The method we carry out at this hospital is perfectly simple, and can be easily understood by the poor people who bring their children to us. The splints consist of simple pieces of wood 2½ in. wide, and long enough to extend 2½ in. beyond the knee-joints above, and level with the soles of the feet below. They are softly padded, and placed on the inner side of the legs.

The exact method of application of these splints is a matter of the utmost importance. Three very firm webbing straps are used; a narrow one below the knee, a narrow one at the ankle, and a wide one, at least 2¼ in. wide, over the most prominent part of the curved bones. The nurse or parent s to be impressed with the importance of this wide strap, for upon its continued tightness and accuracy of adjustment the success of the treatment depends. The buckle of each strap must rest upon the wooden splint, so that the skin should escape injury from their pressure, and the wide strap is to be drawn as tight as the child can bear. It is remarkable how tight this pressure can be tolerated. Not only must this wide centre strap be drawn tight when the splints are applied, but it must be kept tight. Many times during each day may it be necessary to tighten up the centre strap, but this is easily accomplished, and the pressure can thus be kept up. How different from the long bandage from ankle to knee. The latter cannot easily be applied so tightly, and when it gets loose it is much more difficult to tighten again than when dealing with a single strap. The straps at the knee and ankle are merely for keeping the splint in position, and they need not be very tight. The broad strap presses the curved bones towards the splint, that is towards or into a straight position. It is desirable that the patient should wear a stocking or bandage beneath the straps and splint, to prevent injury to the skin from pressure from the edges of the straps. It is absolutely necessary that the splints should be kept on at night as well as during the day. The bones are very elastic, and at first spring back to their old shape when the splints are taken off, but very soon they adapt themselves to the straighter position, and we can always ensure a good result if the pressure be kept up. The splints may, however, be removed whenever necessary for washing, but must be reapplied as soon as possible. As the splints do not project below the shoes, the child can walk about with perfect comfort, and thus need not be kept off his feet. Of course, in cases of severe rickets, where the thigh and other bones are soft and yielding, it may be necessary to restrict the locomotion more or less, but in the majority of cases the child may be allowed to run about freely. After the deformity has been overcome the splints must be worn until by the help of good feeding and the necessary constitutional treatment the patient has become strong, and the tendency in the bones to bend has passed away.

### Wants and Workers.

"NURSE FANNY" has about 50 recent numbers of THE HOSPITAL, and not being desirous of binding them she would like to hear of a nurses' institution or of any poor nurse to whom she could send them.

### Echoes from the Outside World.

### AN OPEN LETTER TO A HOSPITAL NURSE.

Events in South Africa for the last few days have almost been lost sight of because of the great anxiety which surrounds the movements of the Boxers in China. Of course, if things were going badly in the Transvaal, this would not be the case, because, although there are a good many of our countrymen in China, there is not a tithe of the number who are fighting against the Boers. But Lord Roberts seems to have so gathered up the various threads into his hand that we have simply now to wait until he can weave them into the word "Peace," and that he expects the desired end may not be long deferred is apparent from the fact that he has given the Natal Volunteers leave to return to their homes. It is pleasant to read Sir Redvers Buller's statement that they are "followed by the respect and confidence of everyone, and the best wishes of their late comrades." But with regard to China it is because it is so difficult to get an authentic account of what is really taking place that one cannot help fearing that matters are, perhaps, even worse than is really the case. It seems certain that the Boxers—whose watchword seems to be "Kick out the foreigners and kill the Christians," and seem to have been preparing this blow for some time—have vastly increased in numbers since first they started their rebellion, and that they have destroyed the old Roman Catholic Cathedral at Pekin as well as the buildings of the London and American Board of Missions, the Customs House, &c., having at the same time massacred hundreds of native Christians and servants of foreigners. Also that three English and American chapels at Tientsin have been consumed by fire, the work of incendiaries, and that further rioting is feared. But whether Europeans themselves have already fallen victims to the fury of the mob is at present in doubt. The allied squadrons have captured the Taku Forts, but not without the loss, it is said, of six European officers, and 40 marines, and about the same casualties amongst the Japanese. Our own relief column, under Admiral Seymour, is short of supplies, as the railway has been cut and the trains cannot get through. It is stated that they are in the middle of an arid plain, with no food, and hardly any water, and surrounded by a hostile foe. Russia is said to be sending 1,700 troops to assist in restoring order. Japan is despatching between 1,000 and 3,000 men, and America has ordered a regiment of regulars to go over from Manila to the assistance of the Europeans. In the midst of such terrible disorder it is refreshing to read that, notwithstanding all the unfriendly innuendoes which have been written and said, at present at least the Powers seem inclined to unite together in subduing the revolution rather than to take advantage of temporary anarchy to help themselves to fresh territory. It is perhaps reassuring to hear that Li Hung Chang has been summoned to the capital. He at least has no illusions.

As I perused the notices about Mrs. Gladstone, my chief thought was, "How Mr. Gladstone would like to have read what is written about her." For in rare instances, I fear, is marriage such a complete union of souls as it was in the case of the husband and wife who for so many years dwelt together at Hawarden. Some one calls her "a great wife," and no praise, I fancy, would have pleased her more. As a young woman she was spoken of as beautiful and graceful; when I saw her, ten or more years back, the charm of her face lay in its kindliness and its goodness. I do not wonder that she was the recipient of so many confidences, for sympathy was written so large upon her features that one would naturally unburden one's grief to her, feeling sure of help and counsel.

It is said that at the beginning of their married life Mr. Gladstone gave his wife the choice with regard to his political career—to know nothing and to have no responsibility, or to know everything and be bound to secrecy. It is easy to guess her reply, and though she knew every political secret of which her husband had cognisance, she appeared, when "pumped," so quietly and genuinely ignorant that many who have tried to extract at least a hint from her answers, or to betray her into an indiscretion, have gone away almost convinced that the reason she could betray nothing was because she knew nothing herself. One exception, however, I must quote, the little story is so touching. In the early days after Mr. Gladstone had been made a Cabinet Minister, upon one occasion when some of his colleagues were present, his wife made an observation which showed that she knew some matter of confidential importance. At once realising what she had done, she left the room and wrote a note saying how sorry she was, and sent it to Mr. Gladstone by a servant. The reply came back at once: "Dearest C——, don't blame yourself. I don't blame you. It is the only little mistake you ever made. Your affectionate W. E. G." For two years, after having suffered the greatest of losses, Mrs. Gladstone has lived on alone, though surrounded by many who loved her. On Tuesday she was laid once more beside her husband at his express wish, and so, in our most honoured burial-place, beside one of our most gifted men, lies his noble wife until the Resurrection morn. The following telegram was sent by the Queen to Mrs. Gladstone's eldest daughter, the wife of the Dean of Lincoln: "Pray accept my sincerest expression of sympathy and regret at your dear mother's death, whose invariable kindness to me for so many years I shall ever gratefully remember. Pray express my feelings to your brothers and sisters.—V.R.I." At the funeral the Marlborough House wreath bore a touching message of sympathy in the handwriting of the Princess of Wales:—

"In memory of dear Mrs. Gladstone.
"It is but crossing with a bated breath—
A white set face! a little strip of sea—
To find the loved ones waiting on the shore,
More beautiful, more precious than before."

A great sensation was caused by the announcement of a terrible collision at Slough station on Saturday afternoon, between the West of England express and a local train. I do not remember a railway accident involving at least five deaths and injuries more or less severe to upwards of a hundred individuals occurring within such a short distance of the metropolis. The irony of the event was that most of the sufferers were in the train which no one would have supposed could be in any danger. It is a blessing that we do not, as a rule, think of accidents when we are travelling by rail; but the most nervous do not dream of a collision while tickets are being collected. Yet it was at that moment that the disaster at Slough came. How it arose is the subject of inquiry now proceeding. The statement of the Great Western Company is that two signals were against the express, but the engine driver, it seems, asserts that the signal to "slow up" was not given until it was too late to avoid a collision. Poor fellow, what must his sufferings be? No wonder he is prostrate with mental distress, because whether he failed to see the signal, or whether, as he says, the brake failed to act, his position is equally pitiable. All honour to the ticket collector at Slough, who, in order to save a poor passenger from being scalded to death, rushed between him and the escaping steam from the engine, catching the full force on his own back. The worst of a railway accident, even to those not fatally injured, is that sometimes mischief is started which does not betray itself for years. I knew of one young fellow who appeared to have emerged unscathed from a bad collision, and always suffered afterwards from slight paralysis of the hands, and of another who developed brain trouble. In each medical men decided that the cause was mental shock.

### Nursing in a Field Hospital in Natal.

"A MEMBER OF THE ARMY NURSING SERVICE RESERVE" writes: I have had a move, and very pleased I am, too, for it is to a field hospital, nothing but tents. The camp is situated on the veldt, with high mountains all round in the distance, which are just beautiful, and when the sun sets we always watch it—it is such a lovely sight. We are close to Colenso; in fact, the march to the big battle of Colenso was started from this very spot, and the battlefields are literally all around us.

### AT LADYSMITH.

I spent a day lately in Ladysmith and Colenso. Another sister and myself left Chieveley Station when it was quite dark, but when we were getting near Ladysmith the day began to break, and the crimson light over all those battlefields and hills, Umbulwana, Pieters, Monte Cristo, and various other places, besides all the different graves with the little crosses at the head which we noticed all along the line, gave one a deep feeling of awe. We reached Ladysmith at six o'clock; of course it was an awfully early hour to arrive, and there was nothing to do but go for a walk and see the different places which the shells had damaged, and then on to the cemetery, which is outside the little town. We saw Colonel Dick Cunyngham's grave, also Lieutenants Campbell and Dalziel's, and the last resting-place of many others who had been killed or who had died in Ladysmith. The state of the place is terrible. You never smelt anything like it, though, of course, it is worse in some places than others. Then we went to get some breakfast. After some shopping we left for Colenso in a luggage train at half-past twelve. It went very slowly, and we were glad, as it gave us a chance of seeing everything as we went along. The guard pointed out the place where the Intombi Camp stood with its neutral ground and its hospital, and told us where the big Boer guns were placed so that they fired right over the camp into Ladysmith. After we had passed the Klip river came the hills which our men had to take one after another. I have always admired our brave soldiers, but after seeing the country they fought through, one does so perforce more than ever. If only all the people in England who criticised General Buller and said how long he was getting to Ladysmith could see it too! My surprise is that the troops ever did it at all. One of my patients said to me the other day, "Sister, I don't think any other country would have done it; they would have given it up."

### THE BOER TRENCHES.

When we reached Colenso we got out. We walked a couple of miles along the river, and visited the Boer trenches. We saw nothing but a lot of bags, and thinking they might contain bullets I cut one open with my penknife, but I only found dirty river sand and heaps of black beetles, which poured out over my shoes, so I left the bags alone. The scenery around the Tugela is lovely.

### A CUP OF TEA.

On our way back the pangs of hunger and thirst assailed us, as we had had no meal since breakfast, and I asked the sergeant of the guard if there was anywhere we could get a cup of tea. He said no, but the guard had just changed duty, and those soldiers who had come off were making themselves tea, would we accept a cup? Being in uniform, of course he knew who we were. At first we refused, lest we should be robbing them, but as the sergeant looked hurt, and we were both dying for a cup, we marched off with him to where the new bridge was building. The tea was made in a fish kettle, and evidently *boiled* with the tea in it. The men were using their canteens, but they went and hunted up an enamelled cup for us. Having fasted so long, and the tea being so strong, it did us more harm than good, and I do not know which of us felt the worst. It was so kind of the soldiers to give it us, and they seemed so thoroughly pleased to do so that I felt quite a wretch to be so bad after it. We ended our journey in a luggage train again, dead tired, but quite pleased with our day.

### THE CASES.

Now to tell you a little of the nursing. We have hundreds of tents here, some marquees holding as many as seven patients. They are chiefly enteric and dysentery cases. One week we had nearly 250 enterics, and some of them so bad. The "dysenteries," too, are awful sometimes, and we rarely have less than two deaths a day, and often more. There have been nine deaths of sisters and nurses lately; two who left when I came here have both got enteric, and two doctors from here died of enteric in two months, whilst another doctor and the chaplain have it. It is so sad. Our little cemetery by the railway is growing sadly full. Lord Roberts' son is buried there, and numbers of other officers. Nursing in tents, especially with hæmorrhage, is not easy work. All have to accommodate more than the proper number of patients, so some are on stretchers; but they are quite comfortable, as I slept on a stretcher when first I came here till I could get a bed. The only thing that worried me was thinking about the lots of poor fellows who had been on it. Imagine what your back is like after doing a few dressings, or sponging patients lying on stretchers.

### THE CAMP LIFE.

I like the camp life on the whole, but I am glad it is not the rainy season, or I might change my mind. The nurses who came before me saw most of the fighting from here, and once when the hospital was at Spearman's Camp it was within the range of the Boer guns, and they had to move. We can often hear the firing up here, and can see the range of the Biggarsberg and Bulwana very plainly. It may interest you to know that we are on field rations, and have so much given to us every day, and no shops near to supplement the allowance, but we get on very well. Only, as all our food is cooked outside on a camp fire, if it rains the fire goes out.

### WASHING DIFFICULTIES.

We do our own washing, and you would laugh to see us with our sleeves rolled up and washing away with limited water. We pin our clothes on to the tents to dry as we finish them, and the result is quite ornamental. Of course, we can get Indian boys to do it if we like, or send the things by train, but the boys bring them back rough-dried and rolled up in the tiniest of handkerchiefs; and once when I gave a boy some of mine he walked through the camp holding each article up to view as he counted it. So he had no more from me. We have two pussies here, and a dog which attached itself to the Army Medical Corps when they arrived at Durban. It is therefore called by the name of "Durban."

### To Nurses.

**WE invite contributions from any of our readers, and shall be glad to pay for "Notes on News from the Nursing World," or for articles describing nursing experiences, or dealing with any nursing question from an original point of view. The minimum payment for contributions is 5s., but we welcome interesting contributions of a column, or a page, in length. It may be added that notices of entertainments, presentations, and deaths are not paid for, but, of course, we are always glad to receive them. All rejected manuscripts are returned in due course, and all payments for manuscripts used are made as early as possible at the beginning of each quarter.**

### The Prevention of Tuberculosis.

By an East-End Matron.

On Thursday last a conference organised by the Stepney Charity Organisation Society was held in St. Matthew's Mission Room, Stepney, to consider "The Prevention of Consumption in its Relation to Work Among the Poor." The room was crowded, the members of the meeting consisting of East end workers; and Dr. Hillier (from the National Association for the Prevention of Consumption) gave an able address. Several other doctors and gentlemen spoke, discussing how best to cope with the most disheartening difficulties, such as overcrowding in the dwellings of the poor; the increasing prevalence of artificial feeding of infants; the lack of sanatoria for treating hopeful cases; and the difficulty of meeting the expense of a course of treatment, which, to be effective, must continue over a long period of time. Attention was called to the fact that sanitary inspectors are not permitted to examine common lodging houses after eleven o'clock at night, and the urgent need of a law authorising them to visit at any hour of the day or night, as the overcrowding of these lodging-houses after eleven p.m. no doubt added greatly to the spread of consumption. It is a cause for rejoicing that the influential people of Stepney are seeing the necessity of inquiring into and preparing themselves to battle with an enemy which slays more victims yearly than any other disease; but in a many-sided question like the prevention of consumption so much can be said that in a meeting lasting only an hour or two only a few of the main points can be touched upon.

### The Public-House Danger.

There is one great source of evil which appears to me to be frequently overlooked (though it is possible that this apparent overlooking may be due more to the difficulty of coping with the evil than to any oversight). I allude to the public-houses.

In East London the public-houses probably take up more room than any other class of public buildings, and are also more frequented. Englishmen, as a rule, spit a good deal, and among the poor East-Enders this habit is largely indulged in by both men and women. The streets bear abundant evidence of this fact; and the disgusting condition of the stairs in the blocks of "labourers' dwellings" also bear their witness. Even on the floors of their living rooms many of the people spit with a freedom and lack of decent cleanliness which is appalling.

The publican knows his customers and their habits, and hence the special danger arises, for, wishing to avoid the filthy slipperiness which must be caused by the constant spitting of persons coming in and out, he sands his floor and puts sawdust into the spittoons, so that if they are upset by a careless customer an inconvenient mess is avoided, and the moisture of the expectoration is absorbed. Now, if we consider the history and habits of the tuberculosis bacilli we shall realise what a terrible condition of affairs is produced.

### An Illustration.

Take as an example a very common case. A man, after working in the docks on a hot summer day, goes into a public-house for a pot of beer. An hour before another man has been into the same public-house—a man with a wracking cough—who spits on the floor, or, when the coughing fit is very bad and the expectoration profuse, uses one of the saw-dusted spittoons. The sand and the sawdust have absorbed the moisture from the expectoration, and the current of air produced by the constant passing in and out raised the dust, among which the dried particles of sputum have distributed themselves, and the man entering weary and hot breathes into his lungs the seeds of disease—no less vital because unseen.

A few minutes afterwards a woman enters with a baby in her arms, a thin, pale-faced baby, because the father is often out of work, and so the mother goes to a factory when she can, and the baby must get on as best she may without the food Nature provided her. The publican hands a tankard of beer to the woman, and the baby holds out her thin, little hands and cries for a drink; the mother, admiring her knowingness, places the tankard to her lips, and the child drinks. An hour before the man with the cough had drunk from the same tankard, and the potman had washed the ankard hurriedly in cool water and dried it carelessly, so that on the rim a little moisture from the last drinker's lips till remained. Such cases of infection no doubt occur day stby day.

### Appealing to the Patient.

We learn that for every ten persons that die in England at least one is a victim to tuberculosis, that the disease often continues for many years after the invasion before proving fatal, and that the consumptive is spreading the germs of disease wherever he spits during the whole of that time.

It seems to me that the first thing to do in endeavouring to lessen the spread of tuberculosis is to appeal to the patient himself, giving him simple rules, by the observance of which he may cease to be a source of danger to his fellow-men; but those who have practical experience in this matter find that they are often terribly handicapped by the ignorance or indifference of the patient or his friends.

Consumptives, or, indeed, mankind generally, may be divided into two great classes—the unselfish and the selfish. With the unselfish it is comparatively easy to deal. He dreads being the cause of adding to the troubles or sufferings of others, and for the sake of other people will spare himself no pains. But with the selfish man the case is quite different. He rebels against his fate, and does not see why he should inconvenience himself in the smallest degree for the comfort and safety of anyone else, and he will not do so if he can help it. To such a man there is one argument which will scarcely fail to rouse him to carefulness, and that is the fear of reinfection. If you can once convince a selfish consumptive of the infectiousness of tuberculosis, but that with care and the avoiding of reinfection he is curable, you have done more towards protecting society at large than any other measure would ensure.

### A Few Simple Rules.

I should advise the use of the following simple rules:—

1. Always spit into a pocket spitting-flask or small wide-mouthed bottle, containing a small quantity of liquid disinfectant.
2. Dry the mouth with pieces of rag, which should be burnt at once.
3. Avoid kissing on the lips.
4. Use separate cups, spoons, forks for tuberculous patients, and always scald them after use, using a separate vessel in which to clean them.
5. The patient should always sleep alone.
6. Boil all milk.
7. Cook meat thoroughly.
8. And always have the windows wide open both day and night.

### A Word to Nurses.

The Stepney C.O.S. has by its conference given us an example that we may well be proud to follow; and if all nurses, especially district nurses, would take the trouble to familiarise themselves with the means of preventing the spread of tuberculosis, and would convey to the sick and poor with whom they come in contact the knowledge they have gained, more could be done to lessen this fearful scourge than most of us at all realise. A great deal has, I am sure, already been done by nurses, but there is a great deal yet to do, and no nurse has a right to shrink from the trouble and labour which disseminating her knowledge involves.

### Everybody's Opinion.

[Correspondence on all subjects is invited, but we cannot in any way be responsible for the opinions expressed by our correspondents. No communication can be entertained if the name and address of the correspondent is not given, as a guarantee of good faith but not necessarily for publication, or unless one side of the paper only is written on.]

### ROMAN CATHOLIC NURSES AND GUY'S HOSPITAL.

"A. F. C." writes: Regarding your note on Roman Catholic nurses and Guy's Hospital, may I mention that at a well-known hospital in Liverpool Roman Catholic nurses are not only objected to but are treated slightingly, and are compelled to attend Protestant prayers and services? This is in spite of protests. A nurse seeking admission at the hospital in question, upon stating that she is a Roman Catholic, is told that there are no vacancies. But that this is not always true may be judged from the fact that at one time last year the hospital employed no less than four nurses from a neighbouring institution, paying at the rate of six pounds a week in salaries alone. Cannot this anti-Catholic feeling be stamped out? Catholics, I suppose, make as good nurses as Protestants. They also contribute as largely to hospitals as members of any other church. Then why should they not have an equally fair chance in the nursing world? There are as many sick Catholics, perhaps, as there are Protestants, and undoubtedly they prefer to be nursed by one of their own faith. There are also many Catholic girls desirous of being trained as nurses.

### OUTDOOR UNIFORM.

"Nurse R.," Stretford, writes: I am very pleased to learn that the subject of wearing the outdoor uniform by others than the trained nurse is to be discussed at the next meeting of the R.B.N.A. The subject has occupied my mind very much for some time. I have often spoken about it to both those in the profession and out. All agree that something ought to be done to distinguish between the certificated and the uncertificated nurse. The certificate of efficiency, to my mind, should be the means whereby a nurse may obtain possession of whatever distinction may be decided upon. If an Act of Parliament could be obtained prohibiting its use except by nurses possessing such certificate, I do not think there would be many people willing to run the risk of being stopped by the policeman, and render themselves liable to exposure as impostors. I hope that some plan may be formulated. This only expresses the mind of many nurses I know.

"M. S." writes: The abuse of nurses' uniform at the present day is a subject which everyone must allow deserves careful consideration. The most common abuse, and one calculated to do harm is, I think, that certain nurses—usually probationers, to whom uniform is a novelty—parade the dress at all kinds of places of entertainment. There is nothing which attracts more universal attention than our uniform, and the nurse best known to the "people" is the nurse who is inclined to be "fast," hence the very bad opinion of nurses entertained by some persons. Several suggestions have been made in your columns, but what could be more simple or more effectual than the discontinuance of outdoor uniform? What possible good is it for a nurse to wear a dress, off duty, which proclaims her profession? A policeman or a railway porter wears a uniform because it is necessary that he should be in some way distinctive. There are at the present time matrons who discourage the wearing of outdoor uniform. I should be delighted if it were no more seen.

### A SUBSTITUTE FOR PYJAMAS.

"L. M. R." writes: I fully endorse "Y's" opinion as to the unsuitability of pyjamas for any patients who are very ill and at all helpless. This fact was specially impressed upon me this spring, when I was sent for to nurse an elderly gentleman with bronchitis and pneumonia. A few days "home nursing," during which time he had worn pyjamas day and night, had not added to his comfort. With the assistance of a relative of the patient the garment was made which I now describe, and I found it answer so successfully that possibly some of your readers would like to try it. Some flannel, about a yard in width and of a light shade which will wash well, answers the purpose admirably. Either Vyella or Wolla I should specially recommend. If required in a hurry the flannel may be doubled to the required length—this should be to reach about midway between the knee and ankle. The neck must then be cut out, and one breadth divided in half all the way down the front. Roughly speaking, it is an "elongated" pyjama jacket. With a machine the seams can easily be run up, leaving space enough to put in the sleeve. The front is then faced down on either side, and buttons stitched on at intervals, with narrow white cord or braid on the opposite side to avoid the time taken in making buttonholes. About five buttons I found sufficient, as I did not attempt to fasten the garment the whole way down in front. An ordinary round collar may be put on to finish the neck, and of course a certain amount of consideration as to the patient's build must be taken into account. If the garment be torn up the middle of the back for about 18 to 24 inches it will greatly facilitate attending to the patient, and he will have the cool sheet immediately underneath him. This garment can be easily changed during the sweating stage of pneumonia, when it is frequently necessary to put on dry clothing every two or three hours (it is particularly at this time that the fatigue of changing pyjamas is so distressing to the patient). Three or four of these garments are quite sufficient during a mild attack of pneumonia, and the fact that my patient continued to wear them long after he was convalescent showed that he approved of them.

### THE FASHIONABLE LADIES.

"M. M." writes: I saw a statement in a paper the other day from the pen of a "fashionable lady" who had gone out to the front, in which she said that her experience had given her but a poor opinion of the professional nurse. In reading further, and discovering her reasons for forming this opinion, I could not help being amused. She was evidently, in the first place, shocked at the state of the nurses' health, and declared that most of them needed almost as much care as their patients; but she does not say that they got it, or that they had given up and gone off duty. Now a nurse who has worked in the military hospitals in South Africa for a succession of weeks must show some sign of fatigue, and that the nurses have worked till they have dropped we know from more sources than one. That they should be knocked up seems in this lady's opinion to be a sign of general unfitness. She says they show "want of physique, are thin, delicate, and below the medium height." I wonder if she knows that every nurse selected for a hospital has to pass a medical examination to certify she is physically fit, and that, owing to the enormous number of candidates for admission, it is daily becoming more difficult to get passed. It is no question of pressing people into the service, but of keeping them out, and only those who are constitutionally fitted for the work are selected and passed. Then comes a third objection. The lady goes on to say that the nurses are "almost invariably unmarried, nursing being usually embraced as a profession by those of the female sex who have not succeeded in getting married." I should like to see the hospital that would be well and efficiently worked by a staff of married women! They would surely be neglecting the duties of their calling if they left their homes to attend the sick, for no man can serve two masters. That nursing is taken up only by those who have not succeeded in getting married is as wide of the mark as the notion that it is only taken up with the chance of getting married. Those who think that nursing consists in tucking in the sheets and smoothing the pillow, giving an occasional medicine and reading to the patient, soon discover their mistake when they find themselves face to face with the stern reality. The chance of picking up a husband is, under the circumstances, soon pronounced "too dear at the price," and the idea is quickly abandoned. I have seen ladies, who came to be "trained" because it was the fashionable thing to do, pull a very wry face when they have been handed a broom or a scrubbing-brush, and set to some really hard and distasteful work. They loudly declare that they are taught no nursing at all, and disappear from the scene of action with all possible speed. To be able to do all sorts of work is the backbone of a nurse's training, and fits her for any kind of emergency; especially would it fit her for work such as she gets in the hospitals during war.

### For Reading to the Sick.

"Come unto Me all ye that are weary and heavy laden and I will give you rest."

O Jesus, merciful! bend down
In Thy compassions deep,
As sleepless and alone I lie,
And watch beside me keep.

There is a holier, sweeter rest
Than lulling of this pain,
And a deeper calm than that which sleep
Sheds over heart and brain.

It is the soul's surrendered choice
The settling of the will;
Lying gently on the cross
God's purpose to fulfil.

For this I need Thy presence, Lord,
My hand held close in Thine;
Infuse now through my spirit, faint,
An energy divine.

And sanctify my weakness, Lord;
Nature's extreme distress
Is just the time when it may learn
God's glory to express. —*C. M. Noel.*

**Reading.**

In the inner world of thought and feeling, when the beat of Time is heard no more, when the great still certainty of eternity is felt, felt in moments of crisis or of calm; felt when the sun is setting, and out of the crowd you sit alone. . . . felt when the house is quiet and you stare with sleepless eyes into the darkness, and your life is forced back upon its springs. Then all that comes of this world is seen to be insufficient for the immortal spirit; and "first the kingdom of God and His righteousness" is known to be a condition of peace. . . . Repentance, resistance to sin, a mind lifted in loving and eager thoughts to eternal things, a desire, disciplined and away from mere pleasing; these, surely these, are conditions of the peace of the inner life.

Once resting in God, thy rest need not be broken; no, it need not be broken. Of course there must be frailty; of course faults are only gradually overcome; of course we have perplexities; of course we are subject to sorrow. But the penitent soul maintaining its union with Christ in sacrament and prayer, the brave soul striving honestly, faithfully to do its duty, the persevering soul labouring, it may be laboriously, still to go on, cannot fail to realise, cannot fail to rejoice in the blessing of God's peace.—*Canon Knox Little.*

"They shall lie down in the evening,"—
Softly o'er the ear of age,
Comes that word unto the weary,
In their long, late pilgrimage;
There's an hour of still refreshing
From the burden and the heat,
That shall calm the world-worn spirit,
That shall cool the way-worn feet.

"They shall lie down in the evening,"—
Oh, it rings like chimes of rest,
Stealing towards them in the twilight,
From the dim and dreamy west;
For the noon has felt so sultry,
And the path has seemed so long;
Now the day has hushed its voices,
They can hear the even song.

So they lie down in the evening,
And with none to make afraid;
Looking calmly towards the river
Dimly gleaming through the shade.
Death shall seem but summer darkness,
For the lights of earthly love
There shall merge into the brightness
That is breaking from above. —*A. R. Cousin.*

### Notes and Queries.

The Editor is always willing to answer in this column, without any fee, all reasonable questions, as soon as possible.

But the following rules must be carefully observed:—

1. Every communication must be accompanied by the name and address of the writer.
2. The question must always bear upon nursing, directly or indirectly.

If an answer is required by letter a fee of half-a-crown must be enclosed with the note containing the inquiry.

*Arrowroot.*

(122) Is there a simple and sure way of ascertaining if arrowroot is pure and good, or inferior, and perhaps adulterated? My reason for asking is the great difference between the price charged by chemists and that of shops.—*E. B. C.*

It is a difficult matter to detect the adulteration of arrowroot, as it is almost identical with several other starches.

*Comforts for the Soldiers.*

(123) Will you tell me how I am to send a parcel of woollen goods that I am making for the soldiers in South Africa? I think the things would be useful to the hospital staff. I am sorry to trouble you, but have no idea how to act.—*H. W.*

Write to the Hon. Secretary, the Central British Red Cross Committee, 18, Victoria Street, S.W.

*Nurses' Books.*

(124) As we have lectures here, and some of the words are difficult to spell, could you give me the name of a useful dictionary and the price?—*S. H. Q.*

Probably Miss Honnor Morten's useful little book "The Nurses' Dictionary of Medical Terms and Nursing Treatment" is just what you want. The price is 2s., and it is published by the Scientific Press.

*Poro-plastic Corset.*

(125) How could I dispose of a poro-plastic corset?—*A Reader.*

You could send it to a hospital for the use of a poor patient; or you could advertise it for sale; but how to find a person whom it will fit we cannot tell you.

*Over Forty.*

(126) I shall be glad to know if there is any hospital, children's or otherwise, which takes ladies over 40 as paying probationers for six months' training, and the terms for the same.—*Would-be Probationer.*

You might hear privately or by advertisement of a small hospital willing to give you the training you require, but 40 is above the age limit of most institutions. £1 1s. per week is the usual charge for paying probationers.

*Lupus.*

(127) Can you kindly give me some information about an institution for the treatment of lupus? A Danish doctor is said to have discovered a cure for this disease by means of light.—*Nurse Adela.*

The London Hospital has recently undertaken this form of treatment, the Princess of Wales having presented the necessary instruments.

*Assistant Matron.*

(128) Would you kindly inform me where to look for advertisements of situations as assistant matron in a private asylum? 2. Are these situations obtained only through influence?—*Coventry.*

See the "Asylum News" and the various organs of the County Councils and Local Government Boards. 2. No.

*Mentally Afflicted.*

(129) Can you tell me of any place a lady could be sent to (not an asylum) who has been mentally afflicted for five years? Are there any places where she could be sent upon payment of a small fee? (2) Also are nurses (English) well paid and received in Canada?—*Nurse.*

Persons of unsound mind can be received as single patients under certificate. The After-Care Association for Persons Discharged from Asylums, Church House, Dean's Yard, Westminster, might be able to recommend you a suitable home. (2) See the "Professional Handbook," issued by the Emigrants' Information Office, 31, Broadway, Westminster, W.C.

*Midwifery.*

(130) Will you kindly send me word where one has to go for a midwifery examination? I have seen no account in your valuable paper, nor is it in "Burdett's Directory."—*Nurse M.*

Write to the secretary of the London Obstetrical Society, 20, Hanover Square, W., for full particulars. (2) Yes, it is constantly mentioned in the "Queries," and you will find it from the index of "Burdett's Directory" under the heading "Midwifery and Monthly Nursing."

**Standard Books of Reference.**

"The Nursing Profession: How and Where to Train." 2s. net
"The Nurses' Dictionary of Medical Terms." 2s.
"Burdett's Series of Nursing Text-Books." 1s. each.
"A Handbook for Nurses." (Illustrated.) 5s.
"Nursing: Its Theory and Practice." New Edition. 3s. 6d.
"Helps in Sickness and to Health." Fifteenth Thousand. 5s.
All these are published by The Scientific Press, Ltd., and may be obtained through any bookseller or direct from the publishers, 28 & 29 Southampton Street, London, W.C.

## Figures and Tables

**Fig. 16. f1:**